# Model of influenza virus acidification

**DOI:** 10.1371/journal.pone.0214448

**Published:** 2019-04-04

**Authors:** Ajit Akole, Jason M. Warner

**Affiliations:** St. Luke’s School, New Canaan, Connecticut, United States of America; Centro Nacional de Biotecnologia (CNB-CSIC), SPAIN

## Abstract

Internal acidification of the influenza virus, mediated by the M2 proton channel, is a key step in its life cycle. The interior M1 protein shell dissolves at pH~5.5 to 6.0, allowing the release of vRNA to the cytoplasm upon fusion of the viral envelope with the endosomal membrane. Previous models have described the mechanisms and rate constants of M2-mediated transport but did not describe the kinetics of pH changes inside the virus or consider exterior pH changes due to endosome maturation. Therefore, we developed a mathematical model of M2-mediated virion acidification. We find that ~32,000 protons are required to acidify a typically-sized virion. Predicted acidification kinetics were consistent with published *in vitro* experiments following internal acidification. Finally, we applied the model to the *in vivo* situation. For all rates of endosomal maturation considered, internal acidification lagged ~1 min behind endosomal acidification to pH 6. For slow endosomal maturation requiring several minutes or more, internal and endosomal pH decay together in pseudo-equilibrium to the late endosomal pH~5.0. For fast endosomal maturation (≲2 min), a lag of tens of seconds continued toward the late endosomal pH. Recent experiments suggest *in vivo* maturation is in this “fast” regime where lag is considerable. We predict that internal pH reaches the threshold for M1 shell solvation just before the external pH triggers membrane fusion mediated by the influenza protein hemagglutinin, critical because outward proton diffusion through a single small fusion pore is faster than the collective M2-mediated transport inward.

## Introduction

**Fig 1:**
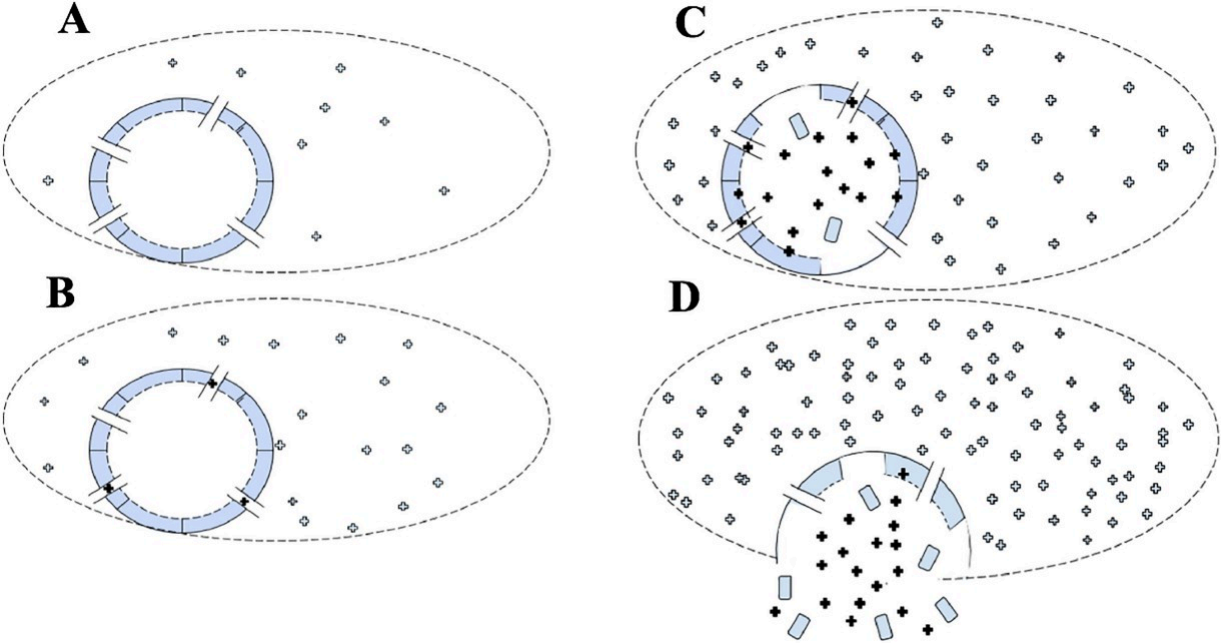
M2-mediated acidification of influenza virus interior. (A) An endocytosed virion bound to the inner leaflet of an early endosome membrane. (B) As the endosome matures and acidifies, protons enter M2 channels and (C) enter the virus interior. (D) Low pH in the virus triggers disassembly of the M1 shell and low pH in the endosome triggers membrane fusion and release of the virus contents into the cytoplasm.

The influenza virus enters cells by endocytosis and takes advantage of low endosomal pH in at least two ways (Fig 1): (i) M2 proton channels in the viral membrane allow acidification of the virus interior, triggering disassembly of the viral core and (ii) the low pH of the endosome triggers conformational changes of the influenza surface protein hemagglutinin (HA) which then drives fusion of the viral and endosomal membranes allowing release of the disassembled contents into the perinuclear cytosol through the fusion pore [1],[2].

Early endosomes begin with essentially neutral pH and their acidification requires ATP-dependent proton pumps in the endosomal membrane to drive cytoplasmic protons inward against an increasing concentration gradient [3]. Some studies indicate that endosomal acidification from neutral conditions to pH~5 requires ~20 minutes [4]. One study found that the endosome spends much of this time moving toward the nucleus with a steady pH near 7, such that most of the acidification takes place over ~2 minutes in the perinuclear region [5]. Viral core acidification is presumably closely tied to, but somewhat delayed from, endosomal acidification, since viral M2 passively permits protons to travel down the concentration gradient from the endosome into the virus. The temporal relationship between endosomal acidification and internal virus acidification has not been established.

The kinetics of proton transport through M2 were studied in controlled *in vitro* experiments where M2 is reconstituted into synthetic vesicles [6],[7]. Proton transport rates of ~10 to ~150 s^-1^ per channel were reported within the pH range 7 to 5, with faster rates driven by lower pH[7]. These are comparable to rates of ~100 to ~400 s^-1^ per channel for influenza virions in an *in vitro* assay at pH 4.6 [8]. Mathematical models for the pH dependence of transport rates were in close agreement with data from the M2 reconstitution experiments [6],[7].

The models of M2 mechanisms are insufficient alone to describe influenza acidification because they do not describe external and internal pH changes or consider how these changes affect proton transport. Notably, external (endosomal) pH decreases gradually over time *in vivo*, in contrast with the large step-like change as *in vitro*. Moreover, internal pH changes are complicated by the fact that the overwhelming majority of transported protons are accepted by the thousands of titratable protein residues in the virus. In this work we develop a mathematical model which, to the best of our knowledge, is the most complete description of influenza acidification.

## Model

Consider an influenza virus surrounded by solution whose acidity pH_ext_(*t*) decreases in time. *In vivo*, this exterior solution is the interior of the endosome whose pH is a controlled by endosomal proton pumps[3]. However, in many *in vitro* experiments, this may be controlled by microfluidic flow [8].

### Proton transport

Leiding *et al* found that proton transport is well described as a two-state process [7]. First, an exterior proton enters the M2 channel and binds to a residue in the lumen according to the rate constant, *k*_*1*_^*+*^. The proton may continue to the virus interior (rate constant *k*_*2*_^*+*^*)* or return to the exterior environment (*k*_*1*_^*-*^*)*. Thus, the transport scheme is

$$M2+H_{\mathrm{ext}}^{+}\;{}_{\overset{\leftarrow }{k_{1}^{-}}}^{\underset{\to }{k_{1}^{+}}}\;M2H^{+}\;{}_{\overset{\leftarrow }{k_{2}^{-}}}^{\underset{\to }{k_{2}^{+}}}\;M2+H_{\mathrm{in}}^{+},$$ Eq 1

where *k*_*2*_^*-*^ is the rate constant for the backward migration of interior protons to the channel.

### Internal proton concentration

Once inside the virus, a proton may contribute to the internal concentration of protons,

$$\left[H_{\mathrm{in}}^{+}\right]=\frac{N_{\mathrm{in}}^{+}}{V},$$ Eq 2

where $N_{in}^{+}$ is the number of free protons in the interior of the virus (volume *V*). However, the virus contains thousands of protein residues that contain so-called titratable groups whose pK_a_ values lie within the pH range of the exterior solution (pH_ext_ = 5 to 7). Hence, free internal protons bind to these residues according to

$$H_{\mathrm{in}}^{+}+Res\;{}_{\overset{\leftarrow }{k_{\mathrm{res}}^{-}}}^{\underset{\to }{k_{\mathrm{res}}^{+}}}\;Res^{+}$$ Eq 3

Since proton binding times are very fast, on the order of 10^−5^ s [9], we make the approximation that interior protons exist in dynamic equilibrium according to

$$K_{a}=\frac{\left[Res\right]\left[H_{\mathrm{in}}^{+}\right]}{\left[Res^{+}\right]}.$$ Eq 4

Since *K*_a_ values are known for each residue, Eq 4 determines the fraction of residues which are protonated at a specific pH. Thus estimating the count of each residue type (see below), the count of protonated residues of each type are calculated then summed to give the total number or protonated residues in the virus.

### Parameter values

Unless otherwise specified, we consider a virus 600 nm in length and 80 nm in diameter [10]. Because the number of M2 channels in a virion does not scale with its size, we allotted 11 M2 channels per virion, the median of the reported 4 to 17 per virion [8]. Table 1 gives rate constants for M2 transport for use in Eq 1. Table 2 shows the number of residues per virion along with the corresponding pK_a_ values for use in Eq 4 [11]. Protein sequences were obtained from the NCBI protein database [12]. M1 proteins are a major source of residues accepting protons, and we estimated 4779 M1 proteins per virion by calculating the number that fit under the membrane using the surface area (24 nm^2^) of each M1 [13],[14]. The counts of all other proteins were obtained from previous studies [8],[15].

**Table 1:** Model rate constants. Values taken from the experiments of Leiding *et al* for M2 channels reconstituted into liposomes.

Rate Constant	Value
*k*_*1*_^*+*^	4.43x10^-8^ M^-1^ s^-1^
*k*_*1*_^*-*^	1000 s^-1^
*k*_*2*_^*+*^	3.05x10^7^ M^-1^ s^-1^
*k*_*2*_^*-*^	68.9 s^-1^

**Table 2:** Titratable residues within the influenza virion. Residues may reside at the c-terminus (c), n-terminus (n), or within the chain (s). For each residue type, the corresponding pKa value (from ref [12]) is given. The total count of each residue in a single virion was estimated by counting each residue type in the sequence of each protein, then multiplying by the copy number of that protein, then summing for all proteins.

Amino Acid (position)	Count	pK_a_
Arg(s)	122073	12.48
Asp(s)	53993	3.86
Cys(s)	19812	8
Glu(s)	113743	4.07
His(s)	38200	6.1
Ile(c)	10	2.32
Lys(c)	5609	2.18
Lys(s)	77678	10.53
Met(n)	5609	9.21
Ser(c)	675	2.21
Tyr(s)	35390	10.07
Val(c)	5	2.29

## Results

### Tens of thousands of protons are required to acidify the virus

We estimate the upper bound of the internal water volume of an influenza virion is 3.1 x 10^−21^ L, assuming it is an empty cylinder 600-nm-long and 80-nm-wide. For such a small water volume, only ~2 protons and ~20 protons are required to reach pH 6 and pH 5, respectively. To determine the number of protons bound to internal protein residues at a given pH, we used the pK_a_ values from Table 2 in Eq 4 to calculate the protonated fraction of each residue, then multiplied the fraction by the corresponding residue count in Table 2. Thus summing over all residues we found ~15,000 residues and ~32,000 residues are protonated at pH 6 and pH 5, respectively. Hence over 99% of protons transported by M2 channels bind virus proteins and do not contribute directly to the pH of the interior solution. Additionally, this means that any error in our estimate of water volume caused a negligible error to the total proton count.

### Acidification kinetics: Model vs. experiment

Next, we assessed the model by comparing its predictions to data from controlled experiments. Ivanovic *et al* [8] followed influenza acidification *in vitro* by loading the virions with fluorescein fluorophores (pK_a_ = 6.4) which are photo-deactivated once protonated. The pH external to the virions was controlled by microfluidic flow replacing the initially neutral solution with an acidic one (pH = 4.5). External acidification was not immediate, but after ~15 seconds of flow, 90% of background fluorescence had dissipated external to the virions. Another ~15 seconds lapsed before the onset of internal fluorescence dissipation, and dissipation occurred over ~34 seconds.

Model parameters (see Tables 1 and 2) were the same as above with the following exceptions. First, we used the mean virus length of their strain, 270 nm [8], to calculate the surface area determining the number of M1 proteins and internal volume. Second, we included 8000 fluorescein molecules in the virions (as estimated by Ivanovic *et al*) which may accept protons transported by M2. Third, we modeled the external acidity using

$$\mathrm{pHext}\left(t\right)=4.5+2.5e^{-t/\tau },$$ Eq 5

where we fit τ = 14.7 s to give pH_ext_ = 5.4 at time t = 15.0 s, corresponding to 90% protonation of the external fluorescein probe. This simple exponential description of external pH gives an initially neutral pH and a final pH of 4.5.

**Fig 2:**
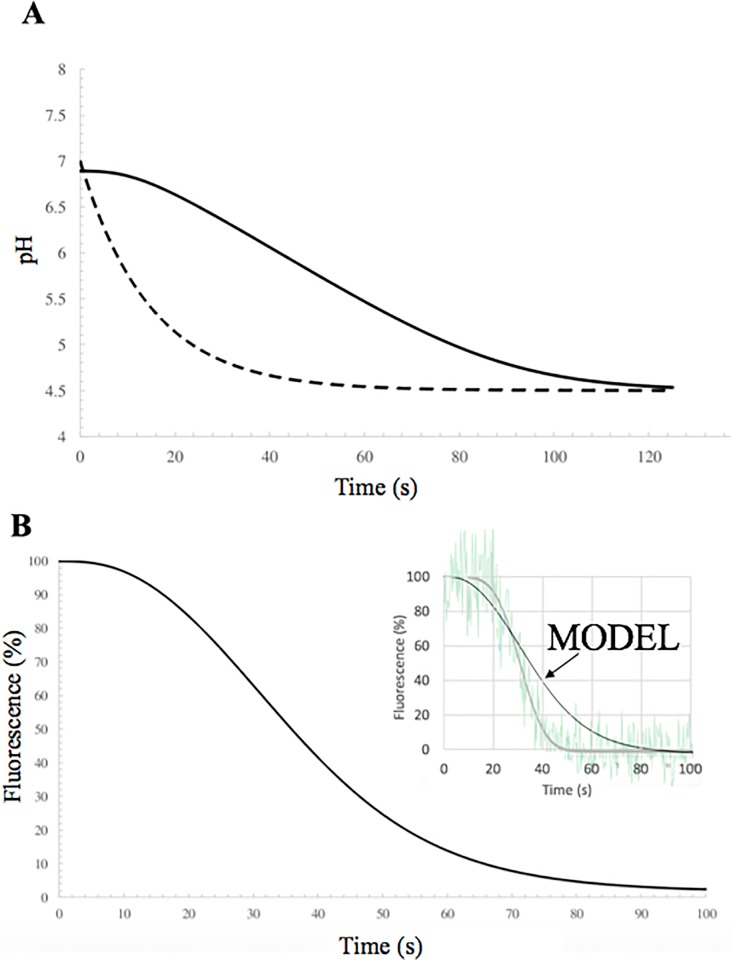
Model predictions for the *in vitro* experiments of Ivanovic *et al*. (A) At time zero, external pH (dashed line) begins a rapid decrease to pH 4.5. Internal pH (solid line) lags behind by as as much as ~1 minute but eventually equilibrates with the external pH after ~2 minutes. (B) In the experiments, acidification was monitored by fluorescein fluorophores loaded into the virions. These photo-deactivate once protonated. Shown here is the predicted fluorescent intensity changes over time. Inset: the representative fluorescence trace from the Ivanovic *et al* publication (raw data in pale green, their fit of the data in gray) is superimposed on the model curve (black). We rescaled the experimental fluorescence (published in arbitrary units) such that the initial fluorescence before acidification and the final background fluorescence correspond to 100% and 0%, respectively.

Model predictions are presented in Fig 2. Internal and external pH are plotted in Fig 2A. Note, in each case, the virus interior reaches pH 6 about ~60 s after the external pH. Ivanovic *et al* published fluorescence measurements rather than direct pH measurements. Hence for more direct comparison we plotted the fraction of fluorescein which remained photo-active during acidification in Fig 2B. The inset of Fig 2B superimposes the fluorescence trace for a single virion of Fig 1C in the Ivanovic *et al* publication (ref. [8]) onto our model curve. The predictions are in reasonable agreement with this representative trace. Additionally, the predictions are in reasonable agreement with the reported averages over all virions in the experiments. Onset of rapid dissipation occurs near t~15 s according to the model, about 15 s faster than the experimental mean. Dissipation is predicted to occur over ~45 s, slightly slower than the experimental mean (34 s). Discrepancies may have resulted from crudity of our estimation of external pH evolution, internal viral protein count, and the number of active M2 channels per virion. In principle these parameters could be fit for exact agreement with the experiments, but with at least three free parameters the fit process would be meaningless

We systematically adjusted model parameters to investigate their effect on acidification kinetics. The rate constants k_1_, k_2_ and r_2_ were each varied by +/-25%, and the resulting time to reach pH 5.7 was determined. Note that these three rate constants determine the other rate constant r_1_ through their relationship with the equilibrium constant (see SI of Leiding et al) such that r_1_ may not be varied independently. The number of M2 channels was varied by +/-27% (+/-3 channels from our above estimate of 11 channels) and the external acidification time was varied by +/-33% (+/-5 s). Table 3 presents the percent change in acidification time (to pH 5.7) from that time (52 s) for the unadjusted parameter set. Also shown is the relative effect of each parameter, which we define as the ratio of the change in acidification time to the change in the parameter value.

**Table 3:** Dependence of acidification kinetics on parameter values. Values taken from the experiments of Leiding *et al* for M2 channels reconstituted into liposomes. Model parameters were individually adjusted by the amount shown in the table, and the resulting change in the time for the internal pH to reach 5.7 was determined by solving the model equations. The relative effect of each parameter adjustment is the ratio of the acidification time change to the parameter change (column 3 divided by column 2) multiplied by 100%.

Parameter	Parameter Change (%)	Acidification Time Change (%)	Relative Effect (%)
k_1_	25	-2.9	12
	-25	4.3	17
k_2_	25	-14.2	57
	-25	23.5	94
r_2_	25	1	4
	-25	-1	4
M2 count	27	-15.4	57
	-27	26.7	98
τ	33	9.4	28
	-33	-9.6	29

The results indicate that k_2_, the rate a proton leaves the M2 channel for the virus interior, and the number of M2 channels have by far the strongest relative effects (57% to 98%), whereas k_1_ had a weak effect (12% to 17%) and r2 had a very weak effect (4%). This is consistent with the observation that, in our numerical calculations, the M2 channels become almost entirely occupied by protons, such that backflow from the virus interior is slow, and that the rate limiting step is forward flow from the channel to the interior. The external acidification time had only a mild effect (28% to 29%) because it occurs sufficiently fast compared to internal acidification.

### Virus size may control acidification time

We noticed that in the experiments of Ivanovic *et al*, the distribution of virus sizes measured using electron microscopy is similar in shape to the distribution of fluorescence dissipation (acidification) times. Hence we used the model to investigate the possibility of a correlation, i.e., whether or not the size of a virion determines its acidification time.

Two model parameters were affected by virus size. First, the volume of the virion interior was readily calculated from its length and width. Second, the number of titratable groups in the virus was adjusted according to size. We adjusted the number of M1 proteins based on the increase in the surface area, as M1 lines the surface of the virus. The number of M2 channels might be expected to increase with virus surface area. However, experimental evidence suggests this is not true [8],[16]. Hence, we assumed the number of M2 channels remained the same, independent of virus size.

**Fig 3:**
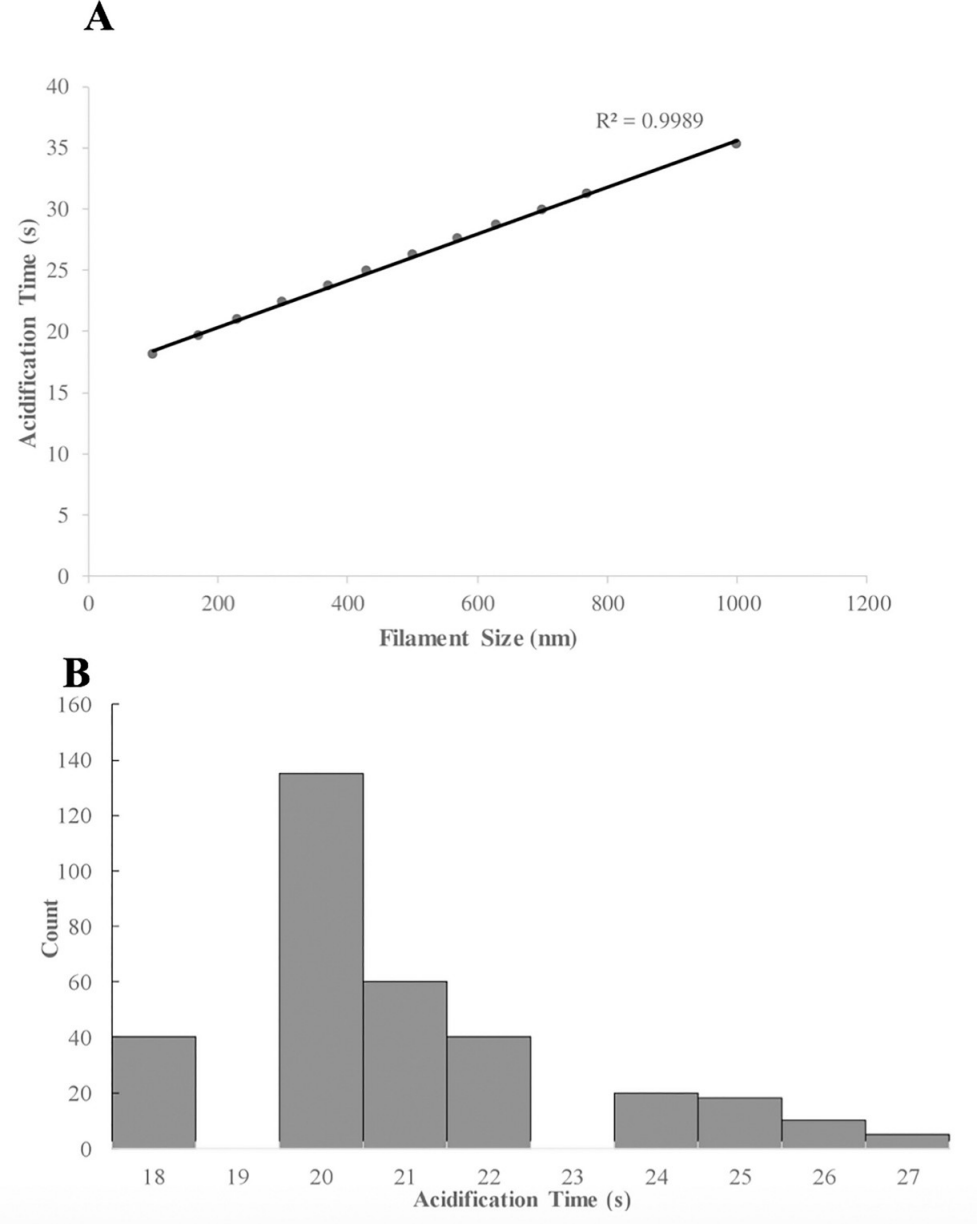
Acidification time increases with virion size. (A) Model predictions indicate that acidification time increases roughly linearly with virion length. (B) Model predictions for the distribution of acidification times for the conditions and distribution of virion lengths of Ivanovic *et al*. The predicted distribution of acidification times is comparable to the experimental distribution.

Fig 3A shows that the predicted acidification time, the time for the viral interior to reach pH 6.4, increases nearly linearly with virus length. This was expected, because the required number of protons increases with virus size but the number of M2 channels does not.

Fig 3B shows the distribution of acidification times predicted by the model for the experiments of Ivanovic *et al*, produced as follows. For the length corresponding to the center of each histogram bin of the experimental length distribution (Fig 4B of ref [8]) we calculated the predicted acidification time. These became the times for the time bins of our distribution, and the height of each of our bins was the count from the experimental length distribution.

The predicted distribution (Fig 3B) peaks at ~20 s and has a half-width of ~5 s. This is comparable to the distribution in Fig 3Bi of Ivanovic *et al*, which peaks at ~30 s and has a half-width of ~10 s. This lends support to the possibility that virus size plays a role in controlling its acidification time. However other factors, such as variations in the M2 content of each virus, may widen the distribution.

### Virion acidification in the endosome

**Fig 4:**
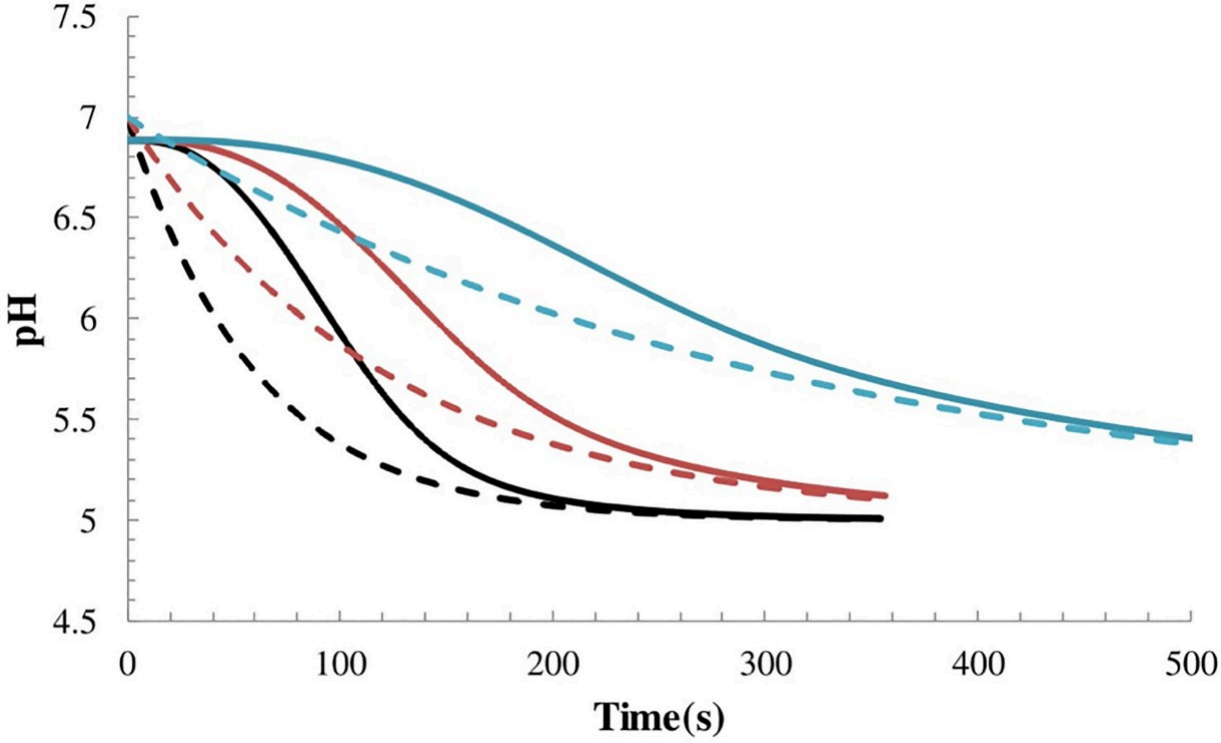
Virion acidification in the endosome. Dashed lines show approximations of endosomal pH evolution for three acidification times: 1 min (black), 2 min (red) and 5 min (blue). Solid lines give predictions for the interior pH of the virion corresponding to each endosomal acidification time. Note that the horizontal axis was trimmed for clarity. For longer times the blue curves approach pH 5 together in pseudoequilibrium.

Next we applied the model to an endocytosed virion. To the best of our knowledge, the quantitative time-evolution of endosomal pH has not been measured. Here we approximate the decay as an exponential, pH_ext_(t) = 5.0+2.0*e*^−*t*/*τ*^, because this is the simplest function describing a pH change from 7 to 5 over a timescale *τ*. There is a wide range of reported timescales for endosomal acidification, from ~2 min to ~20 min [4],[5]. Hence we solved the model for a wide range of acidification timescales. Results are presented in Fig 4. In each case the virion interior reaches pH 6 about 1 min after the endosome but eventually equilibrates with the endosome. For the longest acidification time (blue), the virion pH “catches up” to the the endosome after ~6 min and follows it closely in pseudoequilibrium as pH continues to decay to pH 5.

#### A small fusion pore would rapidly reverse M2-mediated acidification

Next we considered the escape of protons through a fusion pore to the cytoplasm. Fick’s law gives the diffusive flux as *J* = −*D*∇*C* which is driven by the proton concentration gradient ∇*C* and proportional to the diffusivity *D*. Let us approximate the pore as a cylinder of width *w* and length *l*, and assume the concentrations at the pore entrance and pore exit are equal to the bulk concentrations, respectively, inside the virus, *C*_vir_, and outside in the cytoplasm, *C*_cyto_. Thus, the concentration gradient is ∇*C* = (*C*_cyto_−*C*_vir_)/*l*, and using this in Fick’s law and multiplying by the cross-sectional area, *π*(*w*/2)^2^, gives the proton escape rate,

$$\frac{dN_{\mathrm{pore}}^{+}}{dt}=\frac{\pi D\left(C_{\mathrm{cyto}}-C_{\mathrm{vir}}\right)w^{2}}{4l}$$ Eq 6

Proton diffusion is rapid in water with diffusivity *D* = 8×10^−5^ cm^2^/s [17]. Patch clamp experiments reveal HA-mediated fusion pores between an HA-expressing cell and red blood cell were 2 to 5 nm in diameter [18–21]. Using *w = l* = 2 nm in Eq 6 provides a lower bound escape rate of 2800 s^-1^ when the virus interior has pH 6. Lee [22] visualized much larger fusion pores ~15 nm wide and of comparable length between influenza virions and liposomes. For this size the escape rate is 21,000 s^-1^. For comparison, the rate of proton entrance through all M2 channels of the virion is ~230 s^-1^ when the interior pH is 6. Thus the entirety of the range of escape rates is well above the total rate of proton entrance through M2 channels. It is clear that if a fusion pore forms, internal acidification will halt and reverse.

## Discussion

### Timing of low pH-driven M1 shell disassembly relative to membrane fusion

It is apparent that the M1 shell must disassemble, at least in part, to make room for vRNA release [2],[23]. Shell disassembly occurs in the pH range 5.5 to 6 [23],[24]. If a fusion pore is created before sufficient internal acidification, our calculations show that the pore would transport protons much faster than all of the virion’s M2 channels combined; thus internal acidity would quickly be reversed to that of the cytoplasm. Now HA drives fusion at strain-dependent pH levels varying from 4.6 to 6.0 [25]. For the present discussion, let us for simplicity ignore the stochastic nature of shell disassembly and HA activation, and thus approximate that these processes occur quickly at specific pH values, and consider a virion whose HA is activated at pH 5.3 and M1 shell disassembles at pH 5.7 (averages of the corresponding pH ranges above). Does the internal pH reach 5.7 to trigger shell disassembly before the outside reaches pH 5.3 to trigger fusion? Our calculations demonstrate that this is unachievable under any reasonable endosomal conditions (Fig 4). In fact, sufficient internal acidification lags ~1 min behind external acidification. Thus the similar delay of ~1 min for HA-driven pore formation after external acidification to pH 5.3 [26] may be crucial and not a coincidence. Indeed it may be that the each virus strain has tuned itself so that acidification-triggered disassembly of the M1 shell just precedes HA-driven pore formation. More experiments and a comprehensive model combining internal and external acidification kinetics, HA activation, and lipid membrane resistance to topological changes are required to test this hypothesis.

### Possible infection pathway branch point

Fusion pore formation is a stochastic process driven by the action of a few HA molecules, with the width of the distribution of fusion times being comparable to the mean of ~1 min *in vitro* [26]. Since our work suggests internal acidification lags behind HA activation by ~1 min, some but not all virions may stochastically fuse before internal acidification and M1 shell disassembly. These virions would not be infectious as vRNA could not escape. Some facts are consistent with this proposal. For example, it is known that amantadine, a drug blocking M2-mediated proton transport and thus internal acidification, reduces infectivity [22] but that it does not prevent fusion pore creation *in vitro* [8]. Additionally, HA mutations which increase the pH threshold for HA activity reduced infectivity. This could be because HA created fusion pores at higher pH well before internal acidification. Growth of the fusion pore to sufficient size to accommodate vRNA escape may also depend on internal acidification. Recent work [27] suggests that pH-induced M1 shell disassembly may drive expansion of the fusion pore by generating osmotic pressure and membrane tension.

### M2 function in intact virions

In this work we used parameter values for M2-mediated proton transport from *in vitro* experiments using M2-reconstituted liposomes [7]. To measure precise parameter values for M2 function within its viral membrane, presumably one must follow internal pH changes with an internal probe, then use that information to infer proton transport rates. Our work provides a quantitative link between pH and internal proton count and thus could be used for accurate determination of M2 rate parameters.

### Virions as endosomal pH probes

Lakadamyali *et al* [5] used fluorophore-loaded influenza virions to track the time-evolution of endosomal pH over ~2-minute timescales. Our results show a comparable timescale of lag between acidification of the endosome and virion interior. Hence the implicit assumption in ref. [5] that internal virion pH and endosomal pH were equilibrated was somewhat crude. By applying our model, in reverse, to the virion pH data they obtained, one could accurately determine endosomal pH evolution using influenza virions.
